# [Retracted] SHIP1 is targeted by miR-155 in acute myeloid leukemia

**DOI:** 10.3892/or.2026.9129

**Published:** 2026-05-05

**Authors:** Hua Xue, Luo-Ming Hua, Ming Guo, Jian-Min Luo

Oncol Rep 32: 2253–2259, 2014; DOI: 10.3892/or.2014.3435

Subsequently to the publication of the above article, a concerned reader drew the Editor's attention to the fact that the left-hand, outer edges of the β-actin protein blot of lane 1 and the SHIP-1 protein blot for lane 9 in Fig. 1B were hanging over the area representing the grey background of the gels, extending beyond the perceived boundary of these background regions and therefore raising possible concerns about post-acquisition compositing or localized image processing during figure assembly. In addition, in the same figure, the control β-actin western blots shown in Fig. 1B for the lanes 1–8 and 9–16 were apparently the same, albeit the bands in the lower set of blots (for lanes 9-16) were horizontally more compressed.

In view of the potential anomalies described above for the data in Fig. 1B, the Editor of *Oncology Reports* has decided that this paper should be retracted from the Journal on account of a lack of confidence in the presented data. After having been in contact with the authors, they did not accept the decision to retract the paper. The Editor apologizes for any inconvenience caused.

